# Therapeutic potential of targeting the NEDD4L-eEF1A1 axis in cancer therapy: NEDD4L-eEF1A1 axis in regulating tumor angiogenesis

**DOI:** 10.3724/abbs.2025101

**Published:** 2025-06-25

**Authors:** Yan Qin, Xinyue Wang, Minghui Zhao, Yuehui Liu, Huiyi Hou, Tingting Wang, Yuhe Pei, Jingxin Zhang, Zhou Shen, Feixiang Wu, Lishuang Zheng, Jinghua Li, Zhiyu Ni, Jianhong Shi

**Affiliations:** 1 Central Laboratory Affiliated Hospital of Hebei University Baoding 071000 China; 2 Clinical Medical College Hebei University Baoding 071000 China; 3 Hebei Collaborative Innovation Center of Tumor Microecological Metabolism Regulation Baoding 071000 China; 4 Hebei Key Laboratory of Precise Imaging of Inflammation Related Tumors Baoding 071000 China; 5 Department of Radiology Affiliated Hospital of Hebei University Baoding 071000 China; 6 Clinical Medical College Hebei Medical University Shijiazhuang 050000 China; 7 Hepatobiliary Surgery Affiliated Hospital of Hebei University Baoding 071000 China; 8 Affiliated Hospital of Hebei Engineering University Handan 056002 China; 9 Clinical Medical College Hebei University of Engineering Handan 056002 China; 10 Hebei Key Laboratory of Cancer Radiotherapy and Chemotherapy Baoding 071000 China

**Keywords:** NEDD4L, eEF1A1, ubiquitination, angiogenesis, autophagy, proliferation, migration

## Abstract

Abnormal proliferation and migration of endothelial cells are key contributors to tumor angiogenesis. Recent studies have shown that the crucial role of E3 ubiquitin ligase neuronal precursor cell expression developmentally downregulated 4-like (NEDD4L) in tumorigenesis. However, the precise mechanisms by which NEDD4L functions in endothelial cells remain unclear. In this study, we investigate the mechanisms by which NEDD4L influences the function of human umbilical vein endothelial cells (HUVECs) and its effect on tumor angiogenesis. Our results show that NEDD4L overexpression in HUVECs suppresses both cell proliferation and migration. Additionally, we find that the autophagic activity in NEDD4L-overexpressing cells is increased. Proteomic profiling and ubiquitination assays reveal that NEDD4L interacts with eEF1A1, promoting K48-linked ubiquitination-mediated degradation of eEF1A1. This post-translational modification is a key step in the NEDD4L-mediated regulation of autophagy and cellular function. Moreover, we find that loss of endothelial NEDD4L significantly enhances tumor growth and promotes angiogenesis *in vivo*. Overall, NEDD4L plays a crucial role in inhibiting tumor angiogenesis by regulating eEF1A1 ubiquitination and degradation, providing new insights into the NEDD4L-eEF1A1 axis and its potential as a therapeutic target.

## Introduction

Angiogenesis, which involves the formation of new blood vessels from the existing vasculature, is essential for various physiological processes, including tissue regeneration [1]. However, dysregulation of angiogenesis can result in neovascularization, which may worsen or contribute to the progression of several pathological conditions, particularly cancer [ 2, 3] . Emerging evidence indicates that neovascularization is a complex and vital process in oncogenesis, providing essential nutrients and oxygen, facilitating waste removal, and enabling cancer cell metastasis [4]. The proliferation and migration of endothelial cells are crucial for the development of neovascular structures. Moreover, endothelial cells interact bidirectionally with cancer cells, influencing angiogenesis and promoting tumor progression [5]. Consequently, substantial research efforts in recent decades have focused on developing therapeutic strategies to inhibit endothelial cell proliferation and migration in the context of cancer. E3 ubiquitin ligases are a diverse group of enzymes that play pivotal roles in the ubiquitin-proteasome system (UPS), which tags proteins with ubiquitin molecules, targeting them for degradation or modulating their functions via post-translational modifications [ 6, 7] . These ligases are particularly relevant in cancer and tumor angiogenesis, as they regulate the stability and activity of proteins involved in these pathways [ 8– 10] . Additionally, they participate in the regulation of nonprotein targets, such as microRNAs (miRNAs), which influence angiogenic signaling at the post-transcriptional level [ 11, 12] . Therefore, modulating E3 ligase activity—either by altering their function or targeting specific protein degradation—is a promising therapeutic strategy for cancer treatment. The neural precursor cell-expressed developmentally downregulated 4 (NEDD4) family comprises a group of E3 ubiquitin ligases that are crucial for cellular regulation. This family includes key members such as RPF1 (neuronal precursor cell expression developmentally downregulated 4-1), NEDD4L (also known as NEDD4-2, neuronal precursor cell-expressed, developmentally downregulated 4-like), and AIP4 (ITCH/atrophin-1 interacting protein 4) [ 7, 13] . NEDD4L, a member of the NEDD4 family, is an E3 ubiquitin ligase distinguished by a homology to the E6AP C-terminus (HECT) domain, which plays a central role in this highly conserved family. This enzyme regulates several proteins essential for autophagy, cell cycle progression, DNA repair, and antiviral responses [ 14– 16] . Multiple clinical and basic studies have demonstrated an association between NEDD4L and tumor suppression [ 17– 20] . Despite these advancements, the specific functions and mechanisms of NEDD4L in tumor angiogenesis remain poorly understood.

In this study, we investigated the role and mechanisms of NEDD4L in regulating the functions of human umbilical vein endothelial cells (HUVECs). Our results show that NEDD4L overexpression significantly enhances autophagy while simultaneously inhibiting tumor angiogenesis, cell migration, and proliferation in HUVECs via the regulation of eEF1A1 ubiquitination. These findings indicate that NEDD4L may serve as a promising therapeutic target in the context of tumor angiogenesis.

## Materials and Methods

### Experimental model

The animal experiments were approved by the Animal Ethical and Welfare Committee of Hebei University (approval No. IACUC-2023007XR). *NEDD4L* knockout mice on a C57BL/6 background were obtained from Cyagen Biosciences (Suzhou, China). Age-matched endothelial cell-specific *NEDD4L*-knockout mice (NEDD4L ^flox/flox^-Cdh5 ^Cre/wt^, referred to as NEDD4L ^EKO^) and wild-type controls (NEDD4L ^flox/flox^-Cdh5 ^wt/wt^, referred to as NEDD4L ^WT^) were used to establish a tumor-bearing model. NEDD4L-flox mice were crossed with Cdh5-Cre mice to generate a conditional knockout model. All the mice were housed in a specific pathogen-free facility at Hebei University under controlled conditions, including a 12-h light/dark cycle, temperature, and humidity. To establish the tumor-bearing model, 1 × 10 ^6^ cells/mL B16 melanoma cells in 100 μL of serum-free medium were subcutaneously injected into the right flanks of 6-week-old NEDD4L ^WT^ and NEDD4L ^EKO^ mice. Tumor growth was monitored by measuring tumor diameter with callipers every 2 days, and tumor volume was calculated using the following formula: (length × width ^2^)/2. Finally, tumors were harvested for histological analysis and immunofluorescence staining.

### Cell transfection

HUVECs (8000; ScienCell Research Laboratories, Carlsbad, USA) or HEK293T cells (CL-0005; Wuhan Pricella Biotechnology, Wuhan, China) were seeded into 6-well plates and cultured until approximately 80%–90% confluence. Flag-NEDD4L and HA-eEF1A1 expression plasmids or small interfering RNA (siRNA) targeting the human *NEDD4L* gene along with non-specific siRNA (Si-Ctl) was custom designed and synthesized by Gene Pharma (Shanghai, China). These nucleic acids (2 μg) were mixed with 5 μL of Lipofectamine 2000 (12566014; Thermo Fisher Scientific, Waltham, USA) in Opti-MEM (11058021; Thermo Fisher Scientific). The DNA-lipid complexes were incubated for 20 min at room temperature before being added to the cells. After 6 h of incubation at 37°C with 5% CO _2_, the medium was replaced by fresh complete medium, and the cells were further incubated for 24 h. The sequences of all the siRNAs are listed in Supplementary Table S1.

### Adenovirus infection

HUVECs at 90% confluence in 24-well plates were infected with adenovirus serotype 5 (AdV-5) expressing NEDD4L (GenePharma, Shanghai, China) at a multiplicity of infection (MOI) of 40. After washing with phosphate-buffered saline (PBS), the virus was added to the serum-free medium and incubated for 4 h at 37°C in 5% CO _2_. Following infection, the medium was replaced by fresh complete medium, and the cells were incubated for an additional 24 h prior to analysis.

### Protein extraction and mass spectrometry analysis

HUVECs were lysed by sonication three times at 4°C using a high-intensity ultrasonic processor (Scientz Biotechnology, Ningbo, China) in lysis buffer containing 8 M urea and 1% protease inhibitor (P0100; Solarbio, Beijing, China). The lysates were subsequently centrifuged at 12,000 *g* for 10 min at 4°C, after which the supernatants were collected. The protein concentration was determined using a bicinchoninic acid assay kit (P0009; Beyotime, Beijing, China). LC-MS/MS analysis was performed at Sangon Biotech (Shanghai, China), and the results were validated via western blot analysis.

### Co-immunoprecipitation (Co-IP) assays

Co-IP assays were carried out following a previously published protocol [20]. Proteins from HUVECs were immunoprecipitated with 3 μg of specific antibodies (anti-Flag, anti-eEF1A1, anti-NEDD4L, anti-ubiquitin, or anti-HA) for 1 h at 4°C. The antibody-protein complexes were incubated overnight at 4°C with protein A-agarose, followed by centrifugation at 13,400 *g* for 2 min. The pellets were washed five times for 20 min each with IPH buffer at 4°C. The immunoprecipitated proteins were analyzed by SDS-PAGE and western blot analysis using antibodies against eEF1A1 (ab157455; Abcam, Cambridge, UK), NEDD4L (ab46521; Abcam), Flag (A8592; Sigma-Aldrich, St Louis, USA), HA (3F10; Roche Life Science, Basel, Switzerland), and ubiquitin (ET1609-211; HuaAn Biotechnology, Hangzhou, China).

### Immunofluorescence staining

Immunofluorescence staining was performed on 4-μm paraffin sections of mouse tumor tissue and paraformaldehyde-fixed cells. After deparaffinization and rehydration, the slides were blocked with 10% normal goat serum (Y6104L/Y6107L; UElandy, Suzhou, China) for 1 h. HUVECs were permeabilized with 0.1% Triton X-100 in PBS for 30 min. The sections or cells were then incubated overnight at 4°C with the following primary antibodies: anti-CD31 (77699S; Cell Signaling Technology, Boston, USA), anti-NEDD4L (ab46521; Abcam), and anti-eEF1A1 (ab157455; Abcam). Rhodamine and fluorescein-labelled secondary antibodies (S2100; Solarbio) against rabbit and mouse IgG were used. The nuclei were counterstained with 4′,6-diamidino-2-phenylindole (DAPI) (4083S; Cell Signaling Technology), and fluorescence images were captured via an Olympus FV3000 confocal microscope (Tokyo, Japan).

### Western blot analysis

Total protein from cultured cells was extracted using RIPA buffer (R0020; Solarbio) supplemented with a proteinase inhibitor (P1070; Beyotime). Proteins were separated on a 10% SDS-PAGE gel and transferred onto polyester membranes. The membranes were blocked with 5% milk in TBST and then incubated with primary antibodies targeting the following proteins: Beclin1 (ab62557; Abcam), ATG5 (ab108327; Abcam), NEDD4-like E3 ubiquitin protein ligase (ab46521; Abcam), eEF1A1 (ab157455; Abcam), SQSTM1/p62 (PM045; MBL, Tokyo, Japan), AMPK (10929-2-AP; Proteintech, Wuhan, China), p-AMPKα (Thr172) (2531; Cell Signaling Technology), mTOR (66888-1-Ig; Proteintech), p-mTOR (67778-1-Ig; Proteintech), ubiquitin (ET1609-21; HuaAn Biotechnology), HA (3F10; Roche Life Science), Flag (A8592; Sigma-Aldrich), and β-actin (sc-1616-R; Zhongshan Golden Bridge Biotechnology, Beijing, China). HRP-conjugated secondary antibodies (S1002-100; Seracare, Milford, USA) were then applied. Protein detection was performed using chemiluminescence reagent and X-ray films.

### Ubiquitination and cycloheximide (CHX) chase assay

HUVECs were treated with 10 μM MG132 (S2619; Selleck, Shanghai, China). The proteins were extracted from cells using the above described method for immunoprecipitation and western blot analysis. HUVECs were treated with 50 μg/mL CHX for 0, 2, 4, 6, or 8 h, and proteins from cells were extracted for western blot analysis for determination of eEF1A1 half-life.

### RNA isolation and reverse transcription quantitative polymerase chain reaction (RT-qPCR)

Total RNA was extracted from HUVECs using the Superbrilliant 6 min High-Quality RNA Extraction kit (ZS-M11005; Zhongshan Golden Bridge Biotechnology). cDNA synthesis was performed using HiScript III RT SuperMix (R323; Vazyme Biotech, Nanjing, China), and real-time qRT-PCR was conducted with ChamQ Universal SYBR qPCR Master Mix (Q711; Vazyme Biotech). Gene expression levels were normalized to those of *β-actin* and calculated using the 2 ^−ΔΔCT^ method. The primer sequences for *eEF1A1* and *β-actin* are listed in Supplementary Table S2.

### Evaluation of autophagic flux in HUVECs using mRFP-GFP-LC3 double-labelled AAV infection

Autophagic flux in HUVECs infected with mRFP-GFP-LC3 (AP21061002; Hanbio, Shanghai, China) was evaluated as previously described [21]. Briefly, 1 × 10 ^6^ cells/well were infected with Adv-mRFP-GFP-LC3 at an MOI of 80. After the viral medium was replaced by fresh complete medium, the cells were incubated for 24 h to allow expression. The cells were then harvested, washed with PBS, fixed in 4% paraformaldehyde, and imaged using a confocal microscope (FV3000; Olympus, Tokyo, Japan) to assess autophagic flux.

### Wound healing assay

A wound-healing assay was used to assess cell migration. Transfected HUVECs were cultured to 90%–95% confluence in 6-well plates. A scratch was created using a 200-μL pipette tip, followed by three washes with PBS to remove cell debris. Images of the wound area were captured at 0 and 24 h using a Canon EOS 600D camera (Canon, Tokyo, Japan). Semiquantitative analysis was performed using ImageJ software (NIH, Bethesda, USA), and the migration rate was calculated as follows: (initial scratch width at 0 h – scratch width at 24 h) / initial scratch width at 0 h × 100%.

### Transwell migration assay

HUVECs treated with siRNA, AdV-5 expressing NEDD4L, or both were trypsinized and resuspended in serum-free medium at a concentration of 1 × 10 ^6^ cells/mL. Transwell inserts (8 μm pore size) in 24-well plates were precoated with fibronectin. A 100 μL cell suspension was added to the upper chamber, while 800 μL of medium containing 10% FBS (Gibco Life Technologies, Grand Island, USA) was added to the lower chamber. After 24 h at 37°C in 5% CO _2_, non-migrated cells were removed. The inserts were then fixed with methanol and stained with crystal violet (8470; Solarbio). The migrated cells were imaged and counted in five randomly selected fields per insert.

### MTT assay

HUVECs were transfected with siRNA, AdV-5 ex-pressing NEDD4L, or both, and seeded at 5 × 10³ cells per well in 96-well plates. After incubation at 37°C for 24, 48, and 72 h, 20 μL of MTT (M8180; Solarbio) in PBS was added to each well and incubated for 4 h. The MTT was replaced by 100 μL of DMSO to dissolve formazan crystals, and absorbance was measured at 595 nm using a microplate reader (EPOCH; Bio-Tek, Winooski, USA). Cell viability was determined by comparing treated and untreated cell absorbance.

### Endothelial tube formation assay

Pipette tips and 96-well plates were precooled at –20°C. Matrigel (10 mg/mL) (082704T; Mogengel Biotechnology, Xiamen, China) was thawed overnight at 4°C. Once fully liquefied, 50 μL of Matrigel per well was dispensed into prechilled 96-well plates using cold pipette tips. The plates were then incubated at 37°C for 60 min to allow uniform solidification. HUVECs in the logarithmic growth phase were digested to prepare a single-cell suspension. A total of 1 × 10 ^4^ cells were added to each Matrigel-coated well and incubated at 37°C with 5% CO _2_. Tube-like structures were observed, photographed, and recorded under a microscope (Nikon, Tokyo, Japan) after 4 h and 8 h of incubation.

### Endothelial cell isolation from tumor tissue and fluorescence-activated cell sorting analysis

The tumor samples were dissected and rinsed in ice-cold PBS, followed by incubation with 8 mM EDTA at 37°C for 15 min to dissociate the epithelial components. The suspensions were centrifuged at 1200 *g* for 5 min at 4°C, and the resulting cell pellets were enzymatically digested with 0.5 mg/mL Dispase II (04942078001; Roche Life Science) at 37°C for 25 min. This was followed by treatment with a collagenase A/DNase I mixture (1.75 mg/mL collagenase A, 10103586001; Roche Life Science; 0.05 mg/mL Dnase I, D4263; Sigma-Aldrich) at 37°C for 45 min. Single-cell suspensions were stained with CD45-FITC (103107; BioLegend, San Diego, USA) and CD31-PE (566491; BD Biosciences, San Jose, USA) for quantification using a fluorescence-activated cell sorting (FACS) system (CytoFLEX S; Beckman Coulter, Brea, USA). The cells were gated in forward scatter/side scatter (FSC/SSC) plots to exclude debris, and the doublets were eliminated via FSC/FSC gating. CD45 ^–^CD31 ^+^ cells were identified as endothelial cells. Fluorescence-minus-one controls were used to establish gating thresholds and ensure staining specificity.

### Immunohistochemical staining

Immunohistochemical staining was conducted on 4-μm paraffin-embedded tissue sections using a HistostainTM-SP kit (SPN-9001; Zhongshan Golden Bridge Biotechnology). The sections were deparaffinized, rehydrated, and treated with 3% hydrogen peroxide to block peroxidase activity. Nonspecific binding was prevented with 10% goat serum. The sections were incubated overnight at 4°C with primary antibodies against CD31 (77699S; Cell Signaling Technology), α-SMA (14395-1-AP; Proteintech) and VEGFA (HZ-1038; Proteintech) followed by incubation with a horseradish peroxidase-conjugated secondary antibody for 1 h. 3,3′-diaminobenzidine (DAB) (ZLI-9018; Zhongshan Golden Bridge Biotechnology) was used for visualization, and the sections were counterstained with hematoxylin, dehydrated, and mounted. Images were captured with a SPOT Insight 4 Mp CCD camera.

### Statistical analysis

Statistical analysis was performed using GraphPad Prism 8.0. Data are presented as the mean ± standard deviation from at least three independent experiments. Student’s *t* test was used for comparisons between two groups, whereas one-way or repeated measures one-way ANOVA with Tukey’s post hoc test was used for multiple group comparisons. Statistical significance was set at *P*  < 0.05.

## Results

### NEDD4L regulates eEF1A1 protein levels

**Figure 1:**
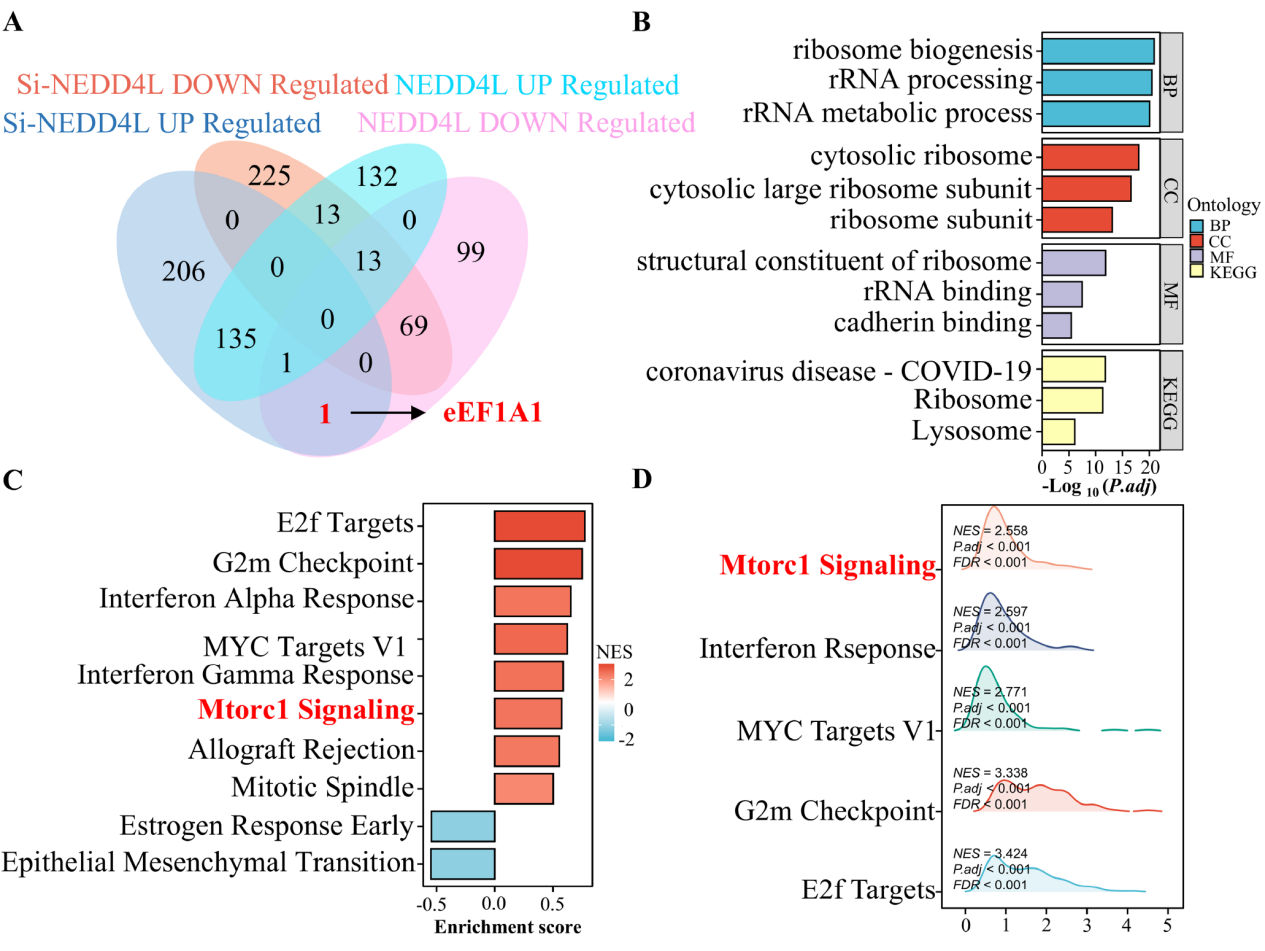
Figure 1 NEDD4L regulates eEF1A1 protein levels, as revealed by mass spectrometry (A–D) Proteomic analysis of differentially expressed proteins between control and NEDD4L-knockdown or NEDD4L-overexpressing HUVECs. The numbers of differentially expressed proteins are shown in the Venn diagram (A), including 294 upregulated and 183 downregulated proteins in the NEDD4L overexpression group and 343 upregulated and 320 downregulated proteins in the NEDD4L knockdown (si-NEDD4L) group. Representative NEDD4L-downregulated and si-NEDD4L-upregulated proteins were subjected to KEGG (B) and GSEA (C,D) functional enrichment analysis. P < 0.05 was considered statistically significant.

NEDD4L, an E3 ubiquitin ligase, mediates both K48-linked [22] and K63-linked [23] ubiquitination, thereby modulating downstream signaling pathways [24]. To identify NEDD4L-regulated substrates in endothelial cells, NEDD4L expression was manipulated in HUVECs, followed by mass spectrometry analysis. This revealed 343 upregulated and 320 downregulated proteins upon *NEDD4L* knockdown and 294 upregulated and 183 downregulated proteins upon NEDD4L overexpression ( Figure 1A). Notably, eEF1A1 was increased with knockdown, or decreased with overexpression, indicating that NEDD4L regulates eEF1A1 levels. A comprehensive bioinformatics analysis, including Kyoto Encyclopedia of Genes and Genomes and gene set enrichment analysis, was performed on these proteins ( Figure 1B–D). Functional enrichment analysis revealed that NEDD4L strongly regulates the cell cycle and the mTOR signaling pathway.

### NEDD4L negatively regulates eEF1A1 protein stability

**Figure 2:**
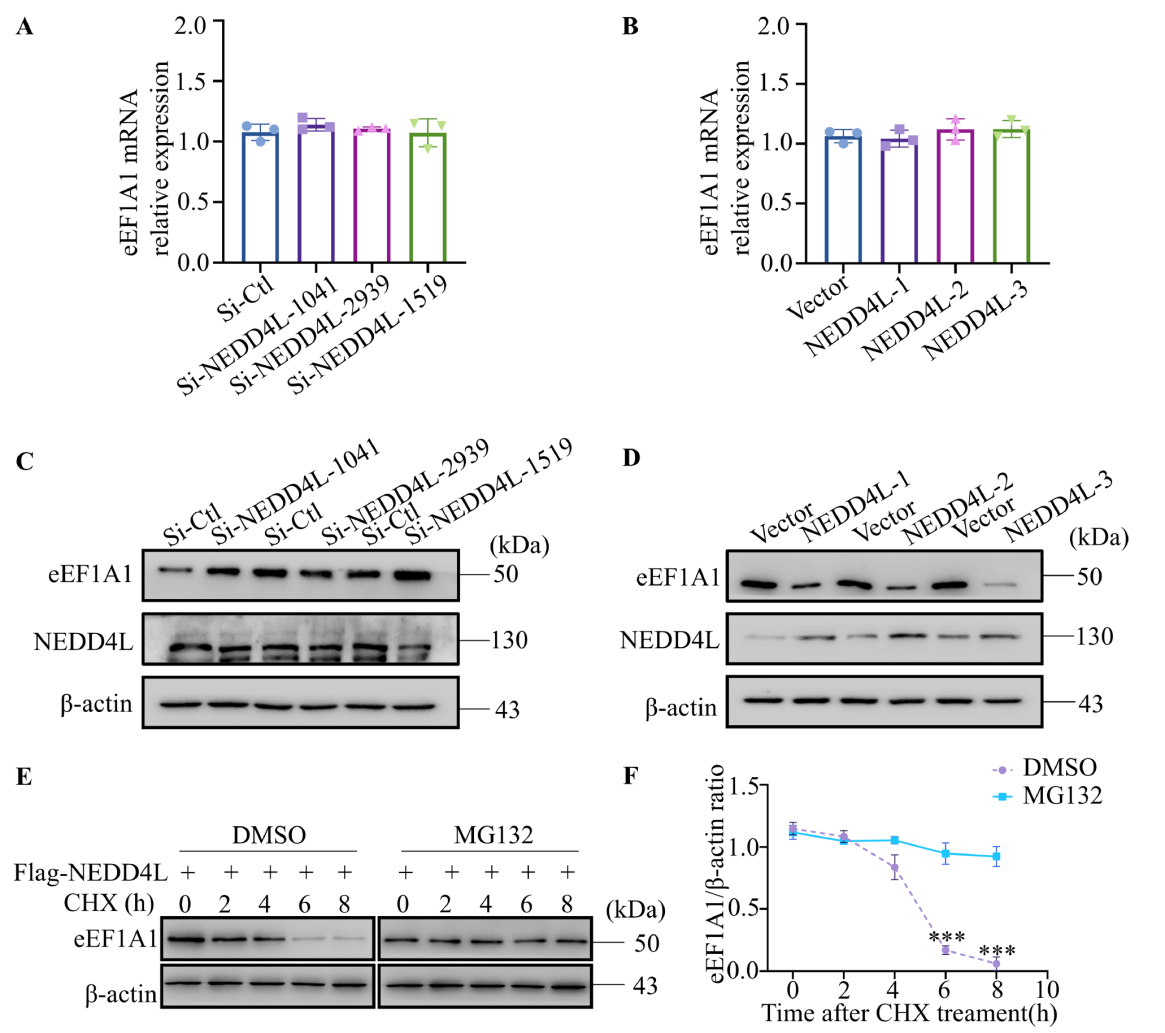
Figure 2 NEDD4L negatively regulates eEF1A1 protein stability HUVECs were transfected with si-NEDD4L or infected with NEDD4L-overexpressing adenovirus, followed by qRT-PCR and western blot analysis. (A,C) Relative mRNA expression levels of eEF1A1 were determined by qRT-PCR and normalized to those of β-actin. (B,D) Western blot analysis was used to detect the protein levels of eEF1A1 (top panel) and NEDD4L (middle panel), with β-actin (bottom panel) as the loading control. (E) HUVECs transfected with Flag-NEDD4L were treated with CHX for 0, 2, 4, 6, or 8 h; eEF1A1 protein expression was assessed by western blot analysis. The band intensities of eEF1A1 proteins were quantified. (F) Relative protein levels of eEF1A1 were analyzed as described. Statistical significance was determined using Student’s t test, ***P < 0.001, n = 3 per group.

To elucidate the effect of NEDD4L on eEF1A1, HUVECs were transfected with si-NEDD4L variants or infected with a NEDD4L-overexpressing adenovirus. qRT-PCR and western blot analysis were used to assess eEF1A1 mRNA and protein expression levels. Neither *NEDD4L* knockdown nor overexpression significantly affected eEF1A1 mRNA expression ( Figure 2A,B). However, *NEDD4L* knockdown led to a marked increase in eEF1A1 protein levels in the si-NEDD4L-1041 and si-NEDD4L-1519 groups, whereas *NEDD4L* overexpression led to a reduction in eEF1A1 protein levels relative to their respective controls ( Figure 2C,D). To assess eEF1A1 protein stability, a CHX chase assay was conducted. CHX treatment resulted in a time-dependent decrease in eEF1A1 protein levels, with an estimated half-life of 6 h in HUVECs. Notably, pretreatment with the proteasome inhibitor MG132 significantly increased eEF1A1 protein stability ( Figure 2E,F), indicating that NEDD4L facilitates eEF1A1 degradation via the proteasome pathway in HUVECs.

### NEDD4L interferes with eEF1A1 and promotes eEF1A1 ubiquitination

**Figure 3:**
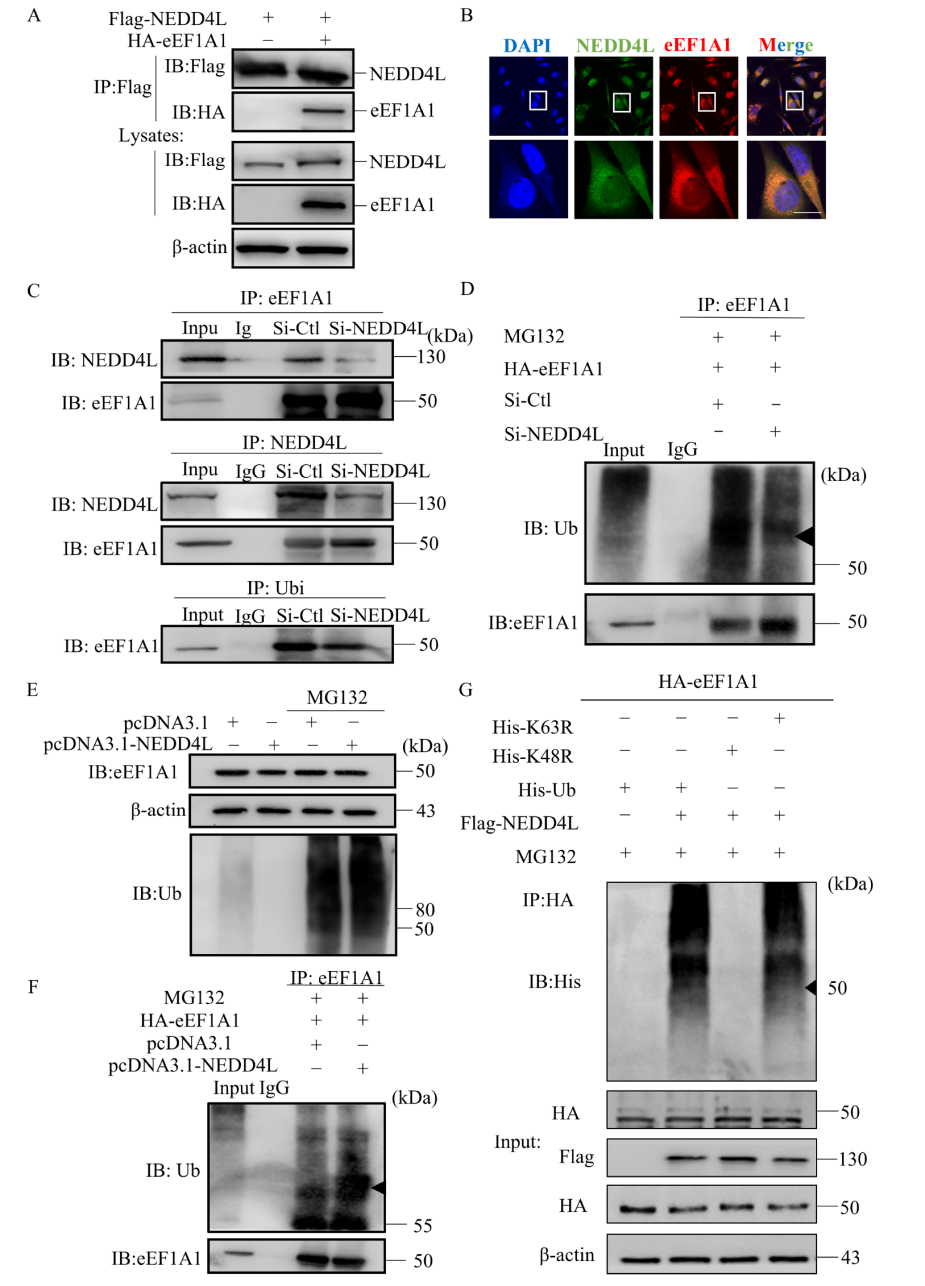
Figure 3 NEDD4L interacts with eEF1A1 and promotes its ubiquitination (A) HEK293T cells were transfected with Flag-NEDD4L and HA-eEF1A1 expression plasmids for 24 h. Cell lysates were immunoprecipitated (IP) using an anti-Flag antibody. NEDD4L was detected by immunoblotting (IB) with anti-Flag, and eEF1A1 was detected using anti-HA. The expression levels of NEDD4L and eEF1A1 in whole-cell lysates were confirmed using anti-Flag and anti-HA antibodies. (B) The subcellular localization of NEDD4L and eEF1A1 in HUVECs was examined by immunofluorescence (IF) staining. DAPI was used to stain the nuclei. Green, red, and blue represent NEDD4L, eEF1A1, and nuclei, respectively. Yellow indicates co-localization of NEDD4L and eEF1A1. Scale bar, 25 μm. (C) HUVECs were transfected with si-NEDD4L for 24 h. Whole-cell lysates were subjected to IP with anti-NEDD4L, anti-eEF1A1, or anti-ubiquitin (Ubi) antibodies and analyzed by western blot analysis, with antibodies against eEF1A1 and NEDD4L. (D) HUVECs were co-transfected with si-NEDD4L and HA-eEF1A1 and treated with MG132. Lysates were subjected to IP with anti-eEF1A1, and the ubiquitination of eEF1A1 was analyzed using anti-eEF1A1 and anti-Ub antibodies. (E) HUVECs were transfected with pcDNA3.1-NEDD4L and/or treated with MG132. Lysates were subjected to IP with anti-NEDD4L or anti-eEF1A1 antibodies and analyzed by western blot analysis with anti-eEF1A1 and anti-Ubi antibodies. (F) HUVECs were transfected with pcDNA3.1-NEDD4L and HA-eEF1A1 plasmids and treated with MG132. The cell lysates were subjected to IP with anti-eEF1A1, and the precipitates were analyzed by western blot analysis using anti-Ub and anti-eEF1A1 antibodies. (G) HEK293T cells were transfected with ubiquitin mutants (K48 or K63) and treated with MG132. Lysates were immunoprecipitated with anti-HA, followed by immunoblotting with anti-His to assess the types of ubiquitin linkages.

To evaluate the potential role of NEDD4L in promoting the ubiquitination of eEF1A1 via the proteasome pathway, we conducted co-transfection experiments in HEK293T cells with Flag-NEDD4L and HA-eEF1A1 constructs. The presence of HA in the precipitate, as detected using an anti-Flag antibody, indicated an interaction between NEDD4L and eEF1A1 ( Figure 3A). This interaction was further supported by confocal immunofluorescence staining, which revealed the co-localization of NEDD4L and eEF1A1 within the cytoplasm of HUVECs ( Figure 3B). To understand the functional consequences of this interaction, we examined how modulating NEDD4L expression affects the ubiquitination of eEF1A1. The specificity of the NEDD4L-eEF1A1 interaction was validated by a reduced association between the two proteins in cells with *NEDD4L* knockdown ( Figure 3C). Furthermore, silencing of *NEDD4L* with siRNA resulted in decreased ubiquitination and increased stability of eEF1A1, as shown by treatment with the proteasome inhibitor MG132 ( Figure 3D). In contrast, NEDD4L overexpression markedly increased the ubiquitination of eEF1A1, resulting in reduced eEF1A1 protein levels ( Figure 3E,F). These findings indicate that NEDD4L interacts with eEF1A1 and promotes its ubiquitination and degradation. To identify the specific sites involved in NEDD4L ubiquitination, we transfected 293T cells with K48R and K63R ubiquitin mutants and assessed how these mutations influence eEF1A1 ubiquitination. The results revealed that the K48R mutation significantly reduced NEDD4L-mediated ubiquitination of eEF1A1, whereas the K63R mutation had no significant effect ( Figure 3G). Collectively, these data indicate that NEDD4L interacts with eEF1A1 and facilitates K48-linked eEF1A1 ubiquitination.

### NEDD4L enhances autophagy in HUVECs

**Figure 4:**
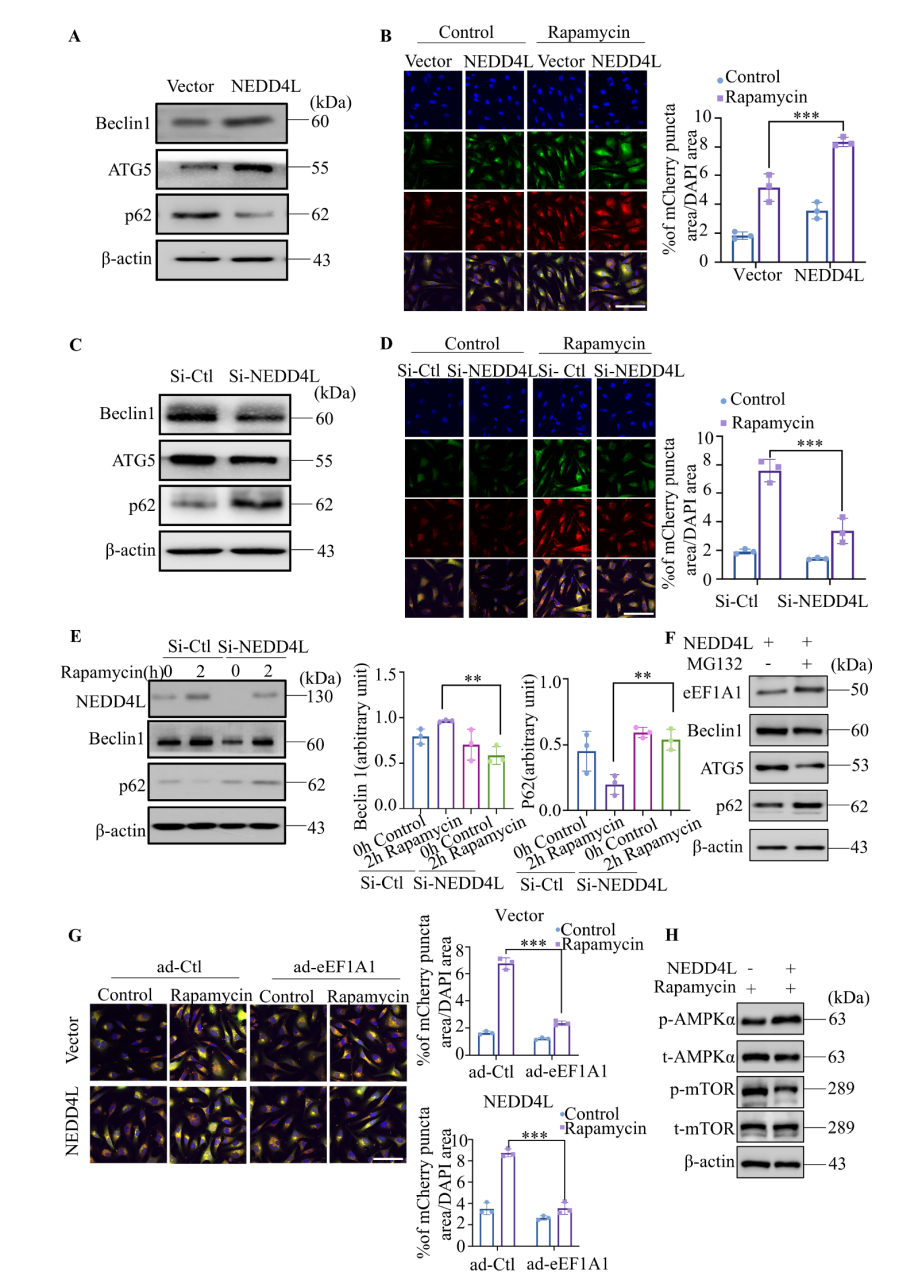
Figure 4 NEDD4L enhances autophagy activity in HUVECs (A,C) HUVECs were transfected with si-NEDD4L or infected with NEDD4L-overexpressing adenovirus. The expression levels of ATG5, p62, and Beclin1 were examined by western blot analysis. (B,D) Cells were transfected with mRFP-GFP-LC3 adenovirus following NEDD4L overexpression or knockdown to monitor autophagic flux. Representative confocal fluorescence microscopy images and quantitative analysis of the GFP-LC3 puncta area (normalized to the DAPI-stained area per cell) are shown. Yellow and red puncta indicate autophagosomes and autolysosomes, respectively. The nuclei were counterstained with DAPI. Data are presented as the mean ± SD of three independent experiments. ***P < 0.001 vs the control group. Scale bar, 100 μm. (E) Western blot analysis was used to assess the expression levels of Beclin1 and p62 in si-NEDD4L-transfected HUVECs with or without 2 h of rapamycin treatment. Band intensities were quantified via ImageJ software, with β-actin used as a loading control. Data are presented as the mean ± SD from three independent experiments. ***P < 0.001 vs the control group. (F) HUVECs were transfected with NEDD4L-overexpressing adenovirus and treated with or without MG132. The expression levels of eEF1A1, ATG5, p62, and Beclin1 were analyzed by western blot analysis. (G) NEDD4L-overexpressing HUVECs stably expressing the GFP-LC3 plasmid were reverse infected with either scramble control adenovirus (ad-Ctl) or eEF1A1-overexpressing adenovirus (ad-eEF1A1). Confocal fluorescence microscopy was used to quantify the GFP-LC3 puncta area. Data are presented as the mean ± SD from at least three independent experiments. ***P < 0.001 vs the control group. Scale bar, 100 μm. (H) HUVECs were transfected with NEDD4L-overexpressing adenovirus and treated with or without MG132. The expression levels of p-AMPKα, t-AMPKα, p-mTOR, and t-mTOR were examined by western blot analysis.

NEDD4L has been recognized as an inducer of autophagy in various cell types [ 21, 25] . In the present study, functional enrichment analysis revealed that NEDD4L significantly regulates the mTOR signaling pathway. To elucidate the role of NEDD4L in modulating autophagy in HUVECs, we overexpressed NEDD4L and observed a significant increase in ATG5 and Beclin1 levels, accompanied by a decrease in p62 protein expression ( Figure 4A). Moreover, HUVECs infected with RFP-GFP-LC3B double-labelled adeno-associated virus (AAV) exhibited increased formation of autophagosomes and autolysosomes upon NEDD4L overexpression, as indicated by increased GFP-LC3 and mCherry puncta signals ( Figure 4B). Conversely, *NEDD4L* knockdown partially reversed rapamycin-induced autophagy in HUVECs, as demonstrated by western blot analysis ( Figure 4C,E) and immunofluorescence staining ( Figure 4D). In HUVECs overexpressing NEDD4L and treated with 10 μM MG132 for 6 h, p62 protein levels increased, whereas Beclin1 and ATG5 levels decreased, indicating that MG132 treatment attenuated the induction of autophagy ( Figure 4F). Notably, the elevated GFP-LC3 and mCherry puncta signals observed in NEDD4L-overexpressing cells treated with rapamycin were significantly diminished upon ad-eEF1A1 treatment, as observed via confocal microscopy ( Figure 4G). These findings indicate that NEDD4L plays a critical role in regulating autophagy in HUVECs and that this effect is dependent on eEF1A1 expression. To explore the mechanism underlying NEDD4L-induced autophagy, we assessed the involvement of the mTOR and AMPK signaling pathways. Compared with control cells, NEDD4L-overexpressing cells treated with rapamycin presented a marked decrease in phosphorylated mTOR (Ser2448) and an increase in phosphorylated AMPKα (Thr172), indicating that NEDD4L promotes autophagy in HUVECs by activating AMPK and inhibiting the mTOR signaling pathway ( Figure 4H).

### NEDD4L affects the proliferation, migration, and invasion ability of HUVECs by regulating eEF1A1

**Figure 5:**
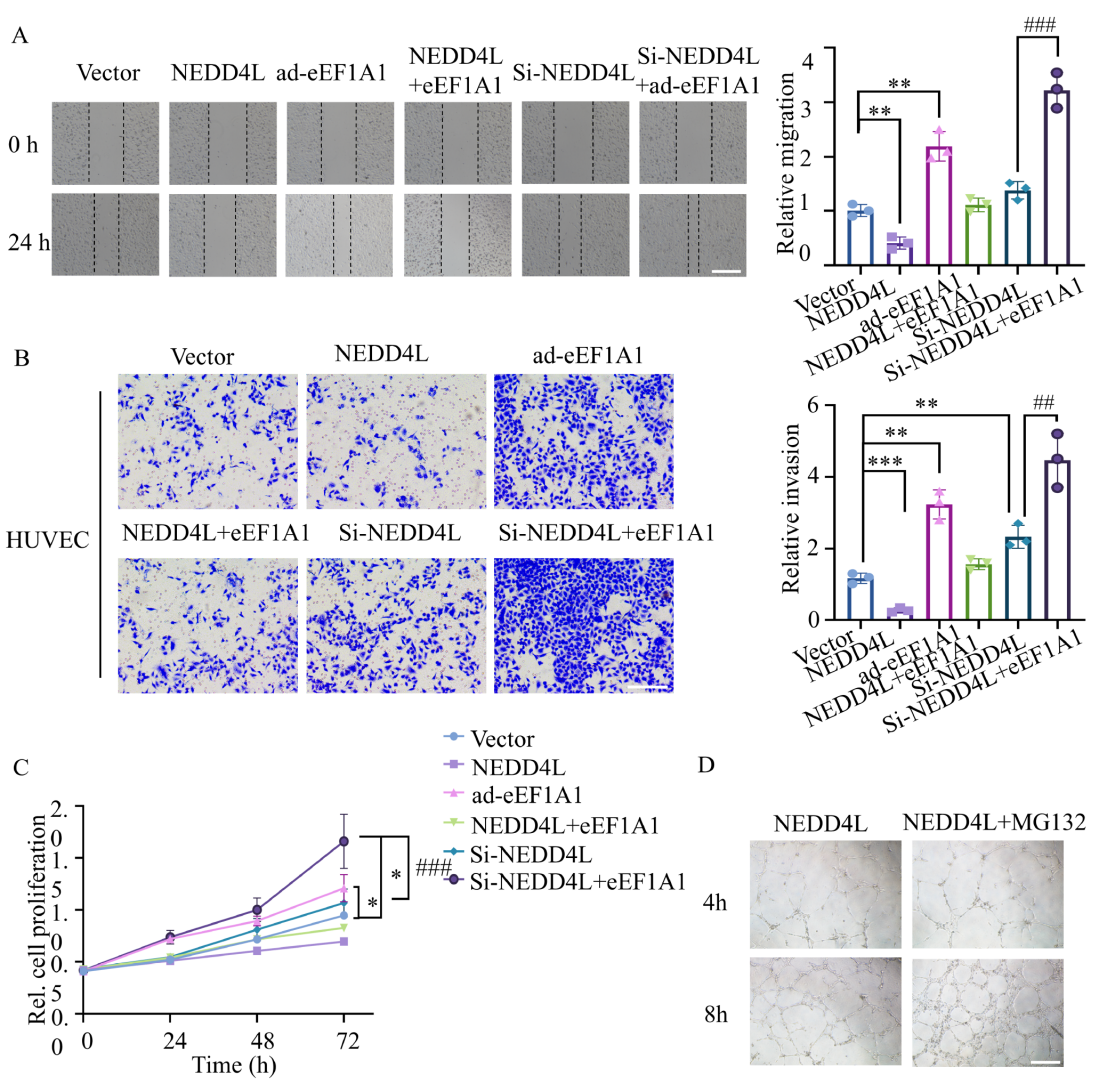
Figure 5 NEDD4L affects the proliferation, migration, and invasion abilities of HUVECs by regulating eEF1A1 HUVECs were treated with siRNA, NEDD4L-overexpressing adenovirus, or a combination of both. (A) Wound-healing assays were conducted to assess the migration of HUVECs. The cells were photographed at 0 and 24 h post-wounding to determine the migration rates into the scratch area. Data are expressed as the mean ± SD from three independent experiments. **P < 0.01, ***P < 0.001 vs the vector group; ### P < 0.001 vs the si-NEDD4L group. Scale bar, 20 μm. (B) Cell invasion was evaluated using Matrigel Transwell assays. Data shown are the quantitative results from three separate experiments. **P < 0.01, ***P < 0.001 vs the vector group; ## P < 0.01 vs the si-NEDD4L group. Scale bar, 10 μm. (C) HUVEC proliferation was determined by MTT assay at 0, 24, 48, and 72 h. A microplate reader was used to measure the optical density at 595 nm (OD595).*P < 0.05 vs the vector group; ### P < 0.001 vs the si-NEDD4L group. (D) Representative images of HUVECs subjected to NEDD4L stimulation with or without MG132 in the endothelial tube formation assay (scale bar, 100 μm). Data are presented as the mean ± SD from three independent experiments.

eEF1A1 has been implicated in the promotion of tumorigenesis [ 26, 27] . Our study further revealed that NEDD4L regulates the cell cycle signaling pathway. We hypothesized that NEDD4L may influence the proliferation, migration, and invasion of endothelial cells by modulating eEF1A1 protein levels. To investigate this, we examined the effects of NEDD4L overexpression and knockdown on the viability, migration, and invasive capacity of HUVECs. As expected, NEDD4L overexpression resulted in a significant reduction in cell migration and invasion ( Figure 5A,B), along with reduced cell growth ( Figure 5C). Conversely, silencing of *NEDD4L* using siRNA increased the proliferation, motility, and invasion of endothelial cells ( Figure 5A–C); however, this effect was reversed upon eEF1A1 overexpression. A Matrigel angiogenesis assay demonstrated that the antiangiogenic effects triggered by eEF1A1 depletion were significantly reversed when proteasome activity was inhibited by MG132 ( Figure 5D). These findings indicate that NEDD4L regulates the proliferation, migration, and antiangiogenic behavior of HUVECs by modulating eEF1A1.

### Conditional deletion of NEDD4L in endothelial cells promotes tumorigenesis and angiogenesis

**Figure 6:**
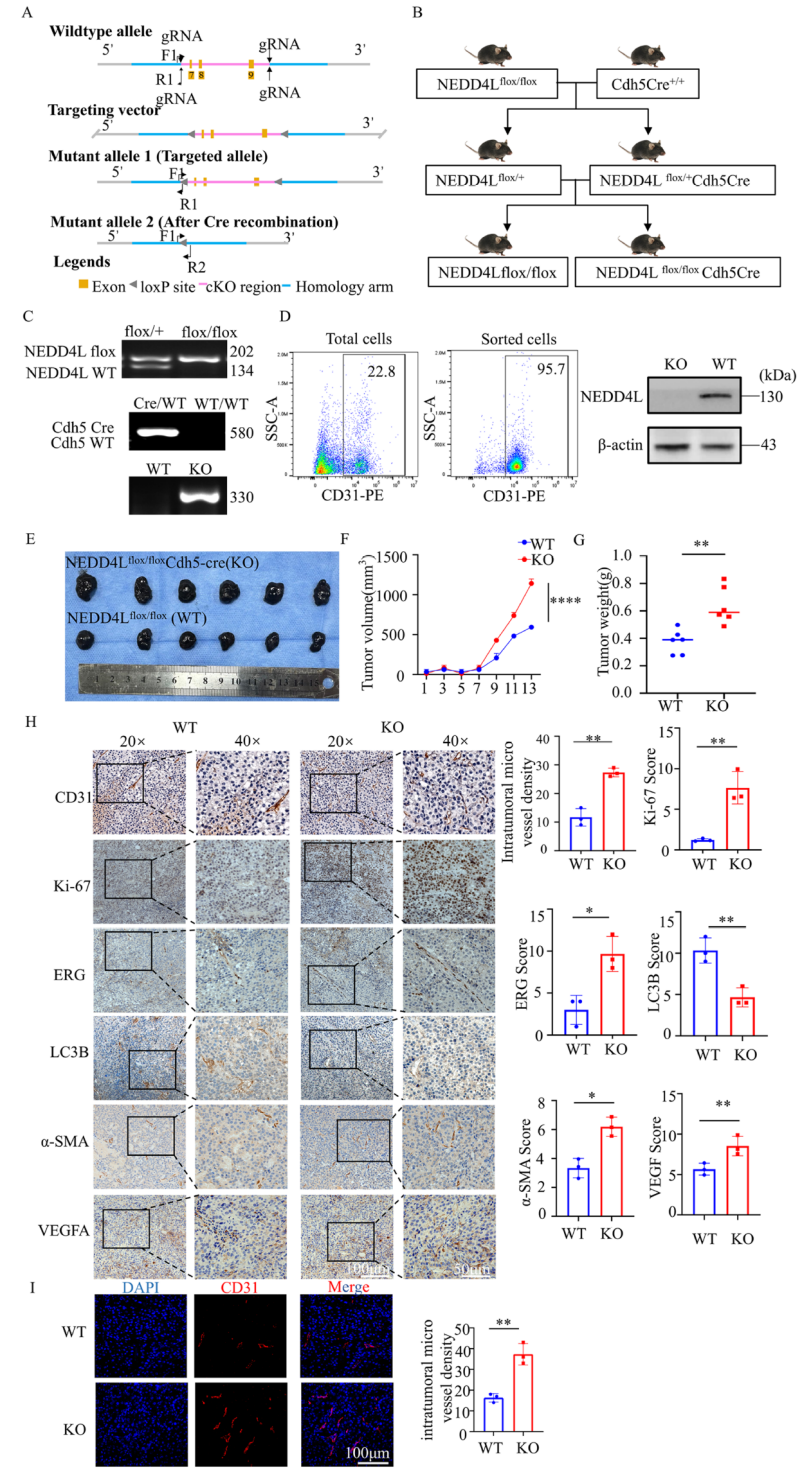
Figure 6 Conditional deletion of NEDD4L in endothelial cells promotes tumorigenesis and angiogenesis (A,B) Schematic representations depicting the homologous recombination targeting strategy to flank the NEDD4L coding region with LoxP sites, thereby creating conditional NEDD4Lflox/flox mice. (C) Ear tissues were collected from 3-week-old mice. DNA was extracted, and after PCR amplification, the genotypes of the mice were detected by agarose gel electrophoresis. (D) Mouse lung endothelial cells were sorted by flow cytometry. Total cellular proteins were extracted, and the expression level of NEDD4L was detected by western blot analysis. (E) Wild-type (WT) mice and mice with conditional deletion of NEDD4L in endothelial cells (KOs) were subcutaneously inoculated with 0.5 × 106/mL B16 melanoma cells. Tumor development was monitored over 2 weeks, after which the tumors were harvested (n = 6 per group). (F) Tumor dimensions were recorded, and volumes were calculated. (G) Tumor weights were measured for each group. (H) Immunohistochemical (IHC) analysis of CD31, Ki-67, ERG, LC3B, α-SMA, and VEGFA expressions in tumor tissues. Images were captured at magnifications of × 20 (scale bar, 100 μm) and × 40 (scale bar, 50 μm). Staining was evaluated and scored in three to five randomly selected high-power fields. Data are expressed as the mean ± SD from three replicate experiments. *P < 0.05, **P < 0.01 vs the WT group. (I) Tumor tissue microvascular density was assessed by immunofluorescence (IF) staining for CD31, with nuclear counterstaining via 4,6-diamidino-2-phenylindole (DAPI). Scale bar, 100 μm. The quantification of microvessel density is presented as the mean ± SD from three independent experiments. **P < 0.01 vs the WT group.

To elucidate the role of NEDD4L in endothelial cells *in vivo*, we generated endothelial cell-specific *NEDD4L*-knockout (KO) mice on a C57BL/6 background. The targeting strategy for the conditional deletion of the *NEDD4L* gene in endothelial cells is illustrated in Figure 6A,B. The genotyping results are presented in Figure 6C. We subsequently established xenograft tumors via the subcutaneous injection of B16 cells to assess whether NEDD4L affects tumorigenesis *in vivo*. Lungs from *NEDD4L*-knockout mice and their wild-type littermates were dissociated, and CD31 ^+^ endothelial cells were isolated via FACS (purity > 95%, confirmed by postsort reanalysis) ( Figure 6D). The NEDD4L protein was subsequently undetectable in CD31 ^+^ cells from knockout mice but was present in CD31 ^+^ cells from wild-type mice. The results revealed significantly accelerated tumor growth and increased tumor size in the KO group compared with the wild-type (WT) group ( Figure 6E–G), indicating that the loss of NEDD4L in endothelial cells promotes tumorigenesis. Immunohistochemical staining was performed to evaluate the proangiogenic role, using markers such as CD31, Ki-67, ERG, and VEGFA, along with α-SMA, to assess functional blood vessels. The results revealed a significant increase in CD31, Ki-67, ERG, and VEGFA expressions as well as increased α-SMA ^+^ vessel coverage in tissues from *NEDD4L*-knockout mice compared with those from controls, indicating that NEDD4L deletion promotes functional angiogenesis. Moreover, autophagy levels—indicated by LC3B—were reduced in the KO group. In contrast, the WT group exhibited the opposite trend ( Figure 6H). Immunofluorescence staining further revealed increased CD31 fluorescence intensity and increased intratumoral microvessel density in the KO group ( Figure 6I), indicating that endothelial cell–specific NEDD4L deficiency accelerates tumor neovascularization.

## Discussion

**Figure 7:**
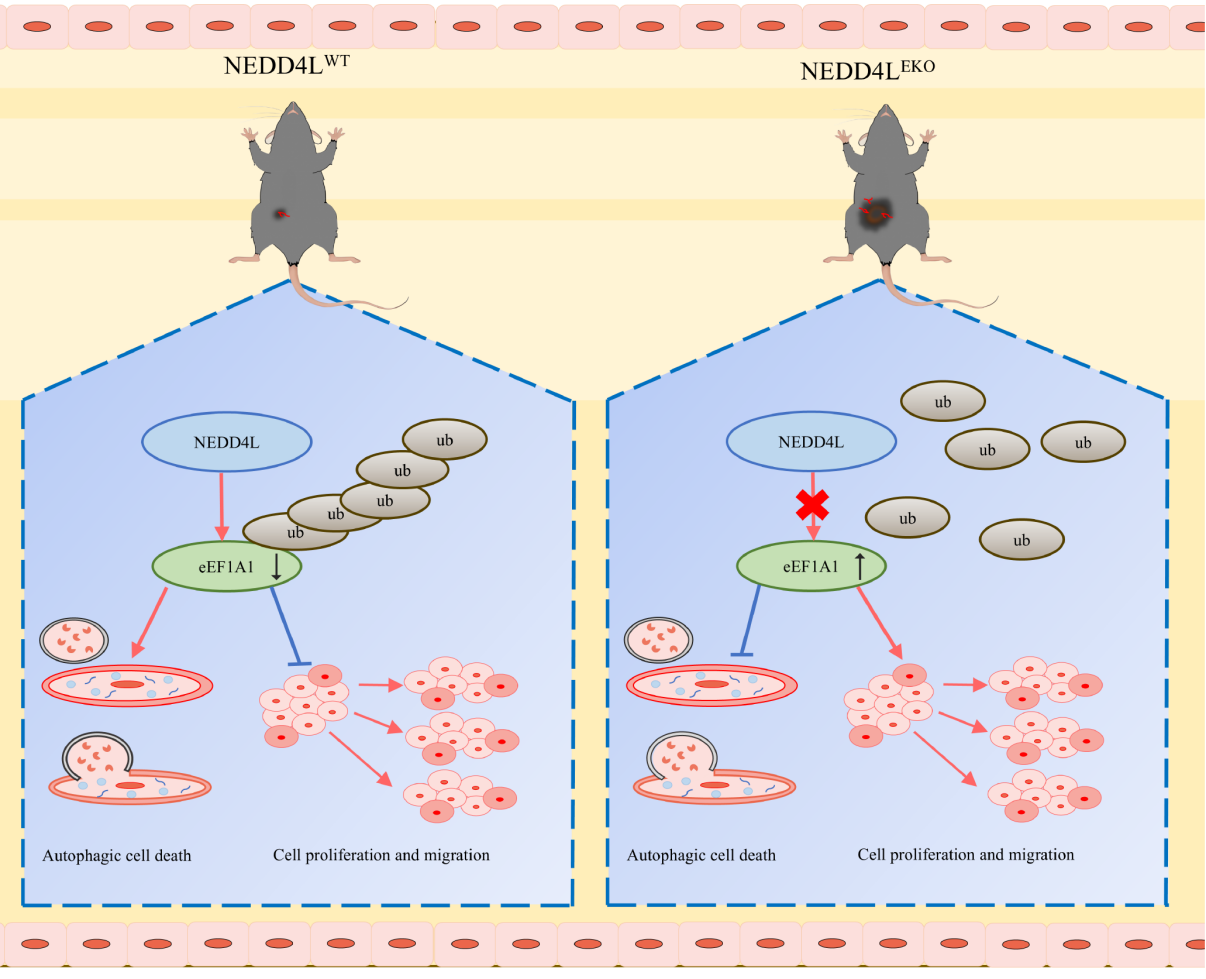
Figure 7 Proposed model for the role of NEDD4L in inhibiting tumor angiogenesis NEDD4L facilitates the ubiquitination and degradation of eEF1A1, leading to the induction of autophagy and the inhibition of tumor angiogenesis, which decreases HUVEC proliferation and migration.

The E3 ubiquitin ligase NEDD4L plays crucial roles in various physiological and pathological conditions, including hypertension, diabetic kidney disease, cancer progression, and cardiovascular disorders [ 19, 28, 29] . However, its role in tumor angiogenesis remains poorly understood. Our study highlights the pivotal role of NEDD4L in regulating endothelial cell activities, particularly autophagy, migration, and proliferation. We found that NEDD4L overexpression induced autophagy and inhibited angiogenic processes by reducing cell proliferation and migration. In contrast, reduced NEDD4L expression enhanced these cellular functions ( Figure 7). Our findings reveal a compelling mechanism by which NEDD4L regulates angiogenesis and endothelial cell behavior by promoting the proteasomal degradation of eEF1A1. These insights into the functions of NEDD4L-regulated HUVECs may contribute to the development of targeted therapies for tumor angiogenesis.

A growing body of evidence implicates NEDD4L in the molecular mechanisms underlying hypertension and cardiovascular diseases, primarily through its role in sodium homeostasis [30]. Our previous study demonstrated that NEDD4L is involved in attenuating inflammation in vascular smooth muscle cells [21]. However, the specific effect of NEDD4L on tumor angiogenesis has not been fully characterized. In our present study, we observed that NEDD4L overexpression in HUVECs significantly inhibited cell proliferation, migration, and angiogenic activity. Conversely, *NEDD4L* knockdown enhanced these functions and promoted functional angiogenesis by modulating VEGFA and α-SMA expression levels, suggesting an antiangiogenic role. This finding contrasts with observations in several cancer types, such as oral squamous cell carcinoma and prostate cancer [18], where NEDD4L overexpression has been shown to suppress proliferation and cell cycle progression, thereby inhibiting tumor growth [ 2, 15, 19, 20, 31] .

NEDD4L, a member of the HECT family of E3 ubiquitin ligases, is recognized for its diverse roles in regulating protein stability and various cellular processes [32]. This enzyme contributes to the progression of multiple diseases by mediating the ubiquitination and subsequent degradation of key protein substrates [ 29, 33] . NEDD4L regulates sodium homeostasis and the epithelial Na ^+^ channel—critical components in hypertension and cardiovascular disease pathogenesis [ 30, 34] . Additionally, NEDD4L has been associated with neurodegenerative disorders by promoting the degradation of α-synuclein [35]. It has also been reported to influence inflammation via the modulation of the NF-κB signaling pathway [ 36, 37] . Furthermore, NEDD4L targets the proto-oncogene Myc for degradation, thereby inhibiting cell viability, cell cycle progression, and glutamine metabolism in esophageal squamous cell carcinoma (ESCC) [38]. In our study, we identified eEF1A1 as a NEDD4L-interacting protein via co-immunoprecipitation and western blot analysis. Our ubiquitination and degradation assays confirmed that eEF1A1 is a direct substrate of NEDD4L. Moreover, we demonstrated that conditional deletion of NEDD4L in endothelial cells promoted tumor growth and angiogenesis. Notably, our data revealed that the enhancement of autophagy and suppression of angiogenesis by NEDD4L are specifically mediated through eEF1A1 degradation. This finding was validated by rescue experiments in which the proteasome inhibitor MG132 was used to inhibit eEF1A1 degradation. Mechanistically, NEDD4L promotes K48-linked ubiquitination of eEF1A1, which directly associates this post-translational modification with proteasomal degradation, in contrast to K63-linked ubiquitination. These findings align with our observations that proteasomal inhibition by MG132 rescues eEF1A1 levels and associated phenotypes. NEDD4L enhances autophagy in HUVECs by activating AMPK and inhibiting the mTOR signaling pathway. Studies on chronic kidney disease have also demonstrated the role of HIF-1α in regulating AMPK activity to limit protein synthesis and activate autophagy [39]. The combined effects of HIF-1α and AMPK activation on the inhibition of protein synthesis via the mTOR pathway are well documented. In our study, we also observed the upregulation of VEGFA, which is regulated by HIF-1α, suggesting that targeting NEDD4L could promote therapeutic angiogenesis. However, whether the AMPK and mTOR signaling pathways are regulated by HIF-1α in HUVECs remains unknown. Collectively, these findings indicate that NEDD4L plays a crucial role in regulating tumorigenesis by catalyzing the K48-linked polyubiquitination of eEF1A1, thereby activating AMPK and inhibiting the mTOR signaling pathway.

Eukaryotic elongation factor 1A1 (eEF1A1) is a crucial component of the eukaryotic translation elongation machinery and plays a key role in protein synthesis [ 40, 41] . In addition to its canonical function, eEF1A1 is involved in various other cellular processes, including cell growth, proliferation, and tumor development [ 27, 42, 43] . Recent studies have highlighted its association with the progression of multiple cancer types [ 44, 45] . In colorectal cancer (CRC), eEF1A1 mRNA and protein levels are significantly elevated in CRC cell lines and tissues compared with their noncancerous counterparts. This overexpression correlates with shorter overall survival among patients with CRC [43]. In HCC, eEF1A1 promotes tumor progression by activating the Akt/mTOR signaling pathway, which is essential for cell growth and survival [46]. The consistent observation of eEF1A1 overexpression across various cancers and its association with poor clinical outcomes underscore the need for further investigation of eEF1A1 as a potential therapeutic target. In our study, we demonstrated a positive correlation between eEF1A1 expression and cell proliferation and survival signaling, whereas NEDD4L overexpression showed an inverse relationship, which was attributed to NEDD4L-mediated ubiquitination and degradation of eEF1A1. However, further studies are warranted to evaluate the therapeutic potential of targeting the NEDD4L-eEF1A1 axis, such as through pharmacological activation of NEDD4L or strategies aimed at inducing eEF1A1 degradation.

In conclusion, the present study provides compelling evidence that NEDD4L acts as a key mediator in promoting autophagy in HUVECs and inhibiting angiogenic activity. Mechanistically, we demonstrated that NEDD4L exerts its effects by directly regulating the ubiquitin-mediated degradation of eEF1A1, thereby activating AMPK and inhibiting the mTOR signaling pathway. These findings suggest the potential of high-throughput screening of small-molecule enhancers that target NEDD4L E3 ligase activity or the use of mTOR inhibitors to induce autophagy. We propose that combining such therapies with the modulation of the NEDD4L-eEF1A1 axis could represent a promising therapeutic strategy for targeting tumor angiogenesis.

## Supporting information

25071Supplementary_Table

## Supplementary Data

Supplementary data is available at *Acta Biochimica et Biophysica Sinica* online.

## References

[1] La Mendola D, Trincavelli ML, Martini C (2022). Angiogenesis in disease. Int J Mol Sci.

[2] Liu S, Guo R, Xu H, Yang J, Luo H, Yeung SCJ, Li K (2023). 14-3-3σ-NEDD4L axis promotes ubiquitination and degradation of HIF-1α in colorectal cancer. Cell Rep.

[3] Carmeliet P, Jain RK (2000). Angiogenesis in cancer and other diseases. Nature.

[4] Fidler, I J. Angiogenesis and cancer metastasis.
*
Cancer J
*, 2000. 6: p.S134–S141. https://pubmed.ncbi.nlm.nih.gov/10803828/.

[5] Luo CK, Chou PH, Ng SK, Lin WY, Wei TT (2022). Cannabinoids orchestrate cross-talk between cancer cells and endothelial cells in colorectal cancer. Cancer Gene Ther.

[6] Sampson C, Wang Q, Otkur W, Zhao H, Lu Y, Liu X, Piao HL (2023). The roles of E3 ubiquitin ligases in cancer progression and targeted therapy. Clin Transl Med.

[7] Tian X, Chen Y, Peng Z, Lin Q, Sun A (2023). NEDD4 E3 ubiquitin ligases: promising biomarkers and therapeutic targets for cancer. Biochem Pharmacol.

[8] Wang M, Zhang Z, Li Z, Zhu Y, Xu C (2023). E3 ubiquitin ligases and deubiquitinases in bladder cancer tumorigenesis and implications for immunotherapies. Front Immunol.

[9] Wang X, Zhang Y, Wu Y, Cheng H, Wang X (2023). The role of E3 ubiquitin ligases and deubiquitinases in bladder cancer development and immunotherapy. Front Immunol.

[10] Liu J, Zhang C, Xu D, Zhang T, Chang CY, Wang J, Liu J (2023). The ubiquitin ligase TRIM21 regulates mutant p53 accumulation and gain of function in cancer. J Clin Invest.

[11] Fan Y, Wang J, Jin W, Sun Y, Xu Y, Wang Y, Liang X (2021). CircNR3C2 promotes HRD1-mediated tumor-suppressive effect via sponging miR-513a-3p in triple-negative breast cancer. Mol Cancer.

[12] Luo Z, Luo H, Fang C, Cheng L, Huang Z, Dai R, Li K (2016). Negative correlation of ITCH E3 ubiquitin ligase and miRNA-106b dictates metastatic progression in pancreatic cancer. Oncotarget.

[13] Wang Z, Hu X, Ye M, Lin M, Chu M, Shen X (2020). NEDD4 E3 ligase: functions and mechanism in human cancer. Semin Cancer Biol.

[14] Wang J, He Z, Liu X, Xu J, Jiang X, Quan G, Jiang J (2022). LINC00941 promotes pancreatic cancer malignancy by interacting with ANXA2 and suppressing NEDD4L-mediated degradation of ANXA2. Cell Death Dis.

[15] Feng R, Li Z, Ge G, Wang C, Jia Y, Ouyang J (2023). NEDD4L represses prostate cancer cell proliferation via modulating PHF8 through the ubiquitin-proteasome pathway. Clin Transl Oncol.

[16] Gao P, Ma X, Yuan M, Yi Y, Liu G, Wen M, Jiang W (2021). E3 ligase Nedd4l promotes antiviral innate immunity by catalyzing K29-linked cysteine ubiquitination of TRAF3. Nat Commun.

[17] Chen S, Li K, Guo J, Chen HN, Ming Y, Jin Y, Xu F (2023). circNEIL3 inhibits tumor metastasis through recruiting the E3 ubiquitin ligase Nedd4L to degrade YBX1. Proc Natl Acad Sci USA.

[18] Zhang G, Zhao X, Liu W (2022). NEDD4L inhibits glycolysis and proliferation of cancer cells in oral squamous cell carcinoma by inducing ENO1 ubiquitination and degradation. Cancer Biol Ther.

[19] Shi Y, Fang N, Wu Y, Xu H, Ning A, Zhang W, Liu Y (2024). NEDD4L mediates ITGB4 ubiquitination and degradation to suppress esophageal carcinoma progression. Cell Commun Signal.

[20] Wu Q, Zhang H, You S, Xu Z, Liu X, Chen X, Zhang W (2023). NEDD4L inhibits migration, invasion, cisplatin resistance and promotes apoptosis of bladder cancer cells by inactivating the p62/Keap1/Nrf2 pathway. Environ Toxicol.

[21] Qin Y, Zheng B, Yang G, Yang H, Zhou J, Yang Z, Zhang X (2020). Salvia miltiorrhiza-derived Sal-miR-58 induces autophagy and attenuates inflammation in vascular smooth muscle cells. Mol Ther Nucleic Acids.

[22] Guo Y, Sun W, Gao W, Li L, Liang Y, Mei Z, Liu B (2022). Long noncoding RNA H19 derived from M2 tumor-associated macrophages promotes bladder cell autophagy via stabilizing ULK1. J Oncol.

[23] Yu J, Sun W, Wang Z, Liang X, Hua F, Li K, Lv X (2019). TRIB3 supports breast cancer stemness by suppressing FOXO1 degradation and enhancing SOX2 transcription. Nat Commun.

[24] Zhang M, Zhang Z, Tian X, Zhang E, Wang Y, Tang J, Zhao J (2023). NEDD4L in human tumors: regulatory mechanisms and dual effects on anti-tumor and pro-tumor. Front Pharmacol.

[25] Huang X, Cao W, Yao S, Chen J, Liu Y, Qu J, Li Y (2022). NEDD4L binds the proteasome and promotes autophagy and bortezomib sensitivity in multiple myeloma. Cell Death Dis.

[26] Lamberti A, Caraglia M, Longo O, Marra M, Abbruzzese A, Arcari P (2004). The translation elongation factor 1A in tumorigenesis, signal transduction and apoptosis: Review article. Amino Acids.

[27] Liu S, Hausmann S, Carlson SM, Fuentes ME, Francis JW, Pillai R, Lofgren SM (2019). METTL13 methylation of eEF1A increases translational output to promote tumorigenesis. Cell.

[28] Li M, Sun G, Wang P, Wang W, Cao K, Song C, Sun Y (2022). Research progress of Nedd4L in cardiovascular diseases. Cell Death Discov.

[29] Han F, Wu S, Dong Y, Liu Y, Sun B, Chen L (2024). Aberrant expression of NEDD4L disrupts mitochondrial homeostasis by downregulating CaMKKβ in diabetic kidney disease. J Transl Med.

[30] Liu H, Lin W, Liu Z, Song Y, Cheng H, An H, Wang X (2021). E3 ubiquitin ligase NEDD4L negatively regulates keratinocyte hyperplasia by promoting GP130 degradation. EMBO Rep.

[31] Gao C, Pang L, Ren C, Ma T (2012). Decreased expression of Nedd4L correlates with poor prognosis in gastric cancer patient. Med Oncol.

[32] Rotin D, Prag G (2024). Physiological functions of the ubiquitin ligases Nedd4-1 and Nedd4-2. Physiology.

[33] Guo XY, Liu TT, Zhu WJ, Liu HT, Zhang GH, Song L, Zhao RN (2022). CircKDM4B suppresses breast cancer progression via the miR-675/NEDD4L axis. Oncogene.

[34] Wright KM, Nathan S, Jiang H, Xia W, Kim HJ, Chakouri N, Nwafor JN (2024). NEDD4L intramolecular interactions regulate its auto and substrate NaV1.5 ubiquitination. J Biol Chem.

[35] Kim TH, Chokkalla AK, Vemuganti R (2021). Deletion of ubiquitin ligase Nedd4l exacerbates ischemic brain damage. J Cereb Blood Flow Metab.

[36] Li H, Wang N, Jiang Y, Wang H, Xin Z, An H, Pan H (2022). E3 ubiquitin ligaseNEDD4L negatively regulates inflammation by promoting ubiquitination ofMEKK2. EMBO Rep.

[37] Zhang X, Wang S, Chong N, Chen D, Shu J, Sun J, Sun Z (2024). GDF-15 alleviates diabetic nephropathy via inhibiting NEDD4L-mediated IKK/NF-κB signalling pathways. Int Immunopharmacol.

[38] Cheng W, Li G, Ye Z, Hu J, Gao L, Jia X, Zhao S (2022). NEDD4L inhibits cell viability, cell cycle progression, and glutamine metabolism in esophageal squamous cell carcinoma via ubiquitination of c-Myc. Acta Biochim Biophys Sin.

[39] Li H, Satriano J, Thomas JL, Miyamoto S, Sharma K, Pastor-Soler NM, Hallows KR (2015). Interactions between HIF-1α and AMPK in the regulation of cellular hypoxia adaptation in chronic kidney disease. Am J Physiol Renal Physiol.

[40] Wilson RB, Kozlov AM, Hatam Tehrani H, Twumasi-Ankrah JS, Chen YJ, Borrelli MJ, Sawyez CG (2024). Elongation factor 1A1 regulates metabolic substrate preference in mammalian cells. J Biol Chem.

[41] Aisha Z, Lei J, Zhang Y, Ma J (2023). EEF1A1 is involved the regulating neuroinflammatory processes in parkinson′s disease. J Integr Neurosci.

[42] Wu W, Xu J, Gao D, Xie Z, Chen W, Li W, Yuan Q (2023). TOPK promotes the growth of esophageal cancer in vitro and in vivo by enhancing YB1/eEF1A1 signal pathway. Cell Death Dis.

[43] Fan A, Zhao X, Liu H, Li D, Guo T, Zhang J, Duan L (2023). eEF1A1 promotes colorectal cancer progression and predicts poor prognosis of patients. Cancer Med.

[44] Jiang H, Zhang Y, Liu B, Yang X, Wang Z, Han M, Li H (2023). Dynamic regulation of eEF1A1 acetylation affects colorectal carcinogenesis. Biol Chem.

[45] Cui H, Li H, Wu H, Du F, Xie X, Zeng S, Zhang Z (2022). A novel 3’tRNA-derived fragment tRF-Val promotes proliferation and inhibits apoptosis by targeting EEF1A1 in gastric cancer. Cell Death Dis.

[46] Zhou P, Chang W, Gong D, Xia J, Chen W, Huang L, Liu R (2023). High dietary fructose promotes hepatocellular carcinoma progression by enhancing O-GlcNAcylation via microbiota-derived acetate. Cell Metab.

