# Supplementary material for: Therapeutic potential of targeting the NEDD4L-eEF1A1 axis in cancer therapy: NEDD4L-eEF1A1 axis in regulating tumor angiogenesis
Source: Acta Biochim Biophys Sin (Shanghai). 2025 Jun 25;58(2):383–95. doi: 10.3724/abbs.2025101 (PMC12900694; doi:10.3724/abbs.2025101)
Supplement: 25071Supplementary_Table [file 25071Supplementary_Table.docx]

**Supplementary Table S1. Sequences of siRNAs used in this study**

| Name | Sequence (5′→3′) |
| --- | --- |
| Si-Ctl | Sense: UUCUCCGAACGUGUCACGUTT |
|  | Antisense: ACGUGACACGUUCGGAGAATT |
| NEDD4L-human1041 | Sense: GGAACUAAGCAGAAGCCUUTT |
|  | Antisense: AAGCCUUCUGCUUAGUUCCTT |
| NEDD4L-human2939 | Sense: GCUUUAAUCGCCUUGACUUTT |
|  | Antisense: AAGUCAAGGCGAUUAAAGCTT |
| NEDD4L-human1519 | Sense: GCUGUGAAAGACACCCUUUTT |
|  | Antisense: AAAGGGUGUCUUUCACAGCTT |

**Supplementary Table S2. Gene-specific primer sequences used in quantitative RT-PCR**

| Gene | Primer sequence (5′→3′) | |
| --- | --- | --- |
| *eEF1A1* | Forward | CCAGGACACAGAGACTTTATCA |
|  | Reverse | CATATCTCTTCTGGCTGTAGGG |
| *β-actin* | Forward | CATGTACGTTGCTATCCAGGC |
|  | Reverse | CTCCTTAATGTCACGCACGAT |
